# A Case of Granulomatous Interstitial Nephritis Associated with *Mycobacterium chimaera* Disseminated Infection

**DOI:** 10.3390/microorganisms13051019

**Published:** 2025-04-29

**Authors:** Martina Cacciapuoti, Maria Mazzitelli, Elena Naso, Maria Loreta De Giorgi, Giovanni Samassa, Valentina Di Vico, Serena Marinello, Lucia Federica Stefanelli, Lorenzo Calò, Annamaria Cattelan, Federico Nalesso

**Affiliations:** 1 Nephrology, Dialysis and Transplantation Unit, University Hospital of Padova, 35128 Padova, Italy; martina.cacciapuoti@aopd.veneto.it (M.C.);; 2 Infectious and Tropical Disease Unit, University Hospital of Padova, 35128 Padova, Italy

**Keywords:** *Mycobacterium chimaera*, disseminated infection, granulomatous interstitial nephritis, cardiac surgery

## Abstract

*Mycobacterium chimaera* infections are becoming increasingly frequent in patients with a history of cardiac surgery. We herein report a case of a patient admitted to the Nephrology Unit of Padua University Hospital with deteriorating kidney function, pancytopenia, hypercalcemia, and respiratory symptoms that emerged seven years after they underwent heart surgery for prosthetic aortic valve replacement. A kidney biopsy revealed non-caseating necrotizing granulomatous interstitial nephritis, which was initially diagnosed as idiopathic granulomatous interstitial nephritis. The patient was treated with intravenous corticosteroids since no active infections, including mycobacterial infections, were detected. The negativity of the *Mycobacterium* molecular test following the kidney biopsy delayed the diagnosis of a *Mycobacterium chimaera* disseminated infection with endocarditis, myositis, cerebral, and kidney involvement, as blood cultures were available only after six weeks. The patient was started on antimicrobial therapy with azithromycin, moxifloxacin, rifampicin, and ethambutol while prednisone was tapered down, leading to an improvement in kidney function, blood count, and blood calcium level. Our case suggests that a *Mycobacterium chimaera* infection should be considered for patients with a history of cardiac surgery and granulomatous interstitial nephritis even in the absence of mycobacteria in a kidney biopsy.

## 1. Introduction

*Mycobacterium chimaera* (MC) belongs to the *Mycobacterium Avium* Complex (MAC). It is an opportunistic human pathogen that colonizes tap water and soil, and it is resistant to many common disinfectants [1]. Although MC is less pathogenic than other MAC species, such as *Mycobacterium avium* or *Mycobacterium intracellulare*, it has been identified as a cause of lung infections in people with chronic obstructive pulmonary disease, cystic fibrosis, bronchiectasis, and cancer and undergoing immunosuppressive therapy.

MC infections usually affect immunocompromised hosts, and those who have undergone cardiac surgery are the most prone to developing this infection [2].

Over the past few decades, there has been an increase in the number of cases of endocarditis and disseminated infections caused by MC, especially after 2015, when the source of the infection was identified in a specific brand of contaminated heater–cooler units (HCUs) for the cardiopulmonary bypass circuit [3] used during cardiothoracic surgery. In 2021, Natanti et al. predicted an increase in MC infections’ prevalence in Italy as clinicians and pathologists’ awareness of this clinical entity increased after the 2015 European outbreak [2]. The Cardiac Surgery Unit of the University Hospital of Padua identified MC in 10.3% of samples collected from 35 HCUs of different manufacturers between January 2017 and May 2022 [4].

The clinical manifestations of MC infection may appear several years (up to 12) after cardiac surgery, raising issues about its classification as a healthcare-associated infection and posing challenges for the diagnostic work up [1]. After endocarditis and cholestatic hepatitis, nephritis is reported as the third most frequent presentation in patients with an MC infection [2]. Kidney involvement usually begins as kidney function impairment due to granulomatous interstitial nephritis (GIN), with or without acid-fast bacteria (AFB) in the granulomas [5,6].

The prognosis for these patients is substantially poor in most cases, with only a 40% 3-year survival rate from the time of diagnosis [1]. There is no standardized treatment, but the combination of azithromycin with ethambutol, rifamycin, and amikacin is the commonly recommended regimen, and antimicrobial susceptibility testing is essential for a targeted therapy. Kidney function impairment prevents combined anti-microbial treatments including amikacin [7], which is historically well-known for its nephrotoxicity.

Although MC disseminated infections have already been described in Italy [1,2,4,8], none of the associated studies reported kidney involvement. Our case report concerns granulomatous interstitial nephritis associated with an MC infection treated at the Nephrology Unit of Padua University Hospital.

The patient provided consent for the publication of his clinical history, information, and images. Moreover, this report was submitted to our Ethics Committee, which approved retrospective data collection.

## 2. Case Report

A 56-year-old gentleman was referred to our tertiary-care hospital (Azienda Ospedale Università di Padova, Padua, Italy) for a sudden and unexplained increase in serum creatinine (sCr) levels from 261 umol/L to 304 umol/L, which occurred over the course of one month and was associated with pancytopenia, hypercalcemia (2.71 mmol/L), and suppressed PTH (<4 pg/mL). The patient also reported respiratory symptoms and presented with sub-nephrotic proteinuria (1.48 g/24 h) and hematuria (112 mg/mL). The patient also had two lesions on his thigh, which were subsequently biopsied.

**Figure 1:**
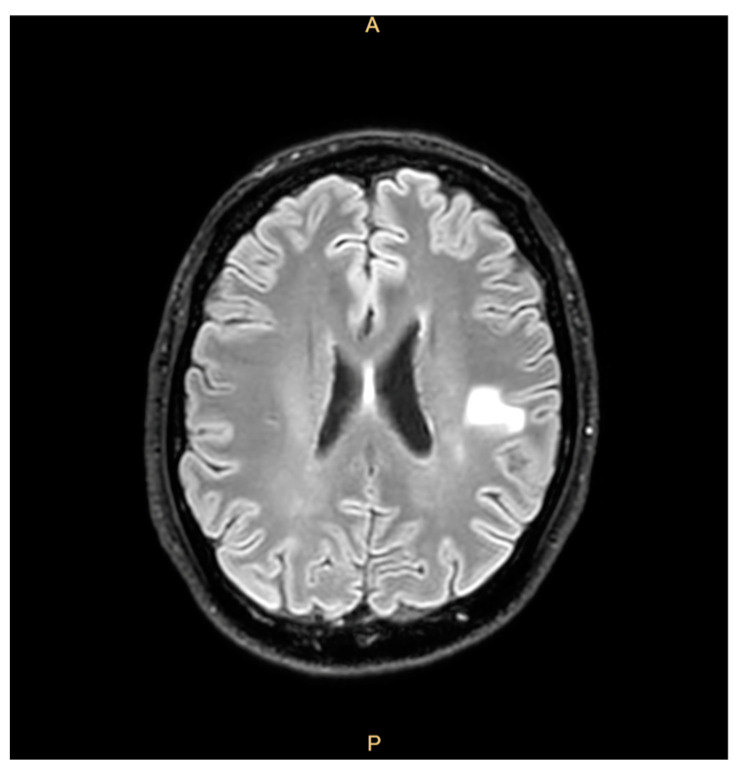
Brain magnetic resonance showing outcomes of hemorrhagic lesions.

The patient had a past medical history of a prosthetic valve replacement 7 years earlier for a severe aortic insufficiency due to a ventricular septal defect (VSD). Four years later, the patient received a recall notification telling him to come to the hospital as part of an investigation promoted by the Veneto Region; this recall concerned all patients who had undergone major cardiac surgery between 2010 and 2017 (https://www.osservatoriomalattierare.it/malattie-rare/malattia-polmonare-da-micobatteri-non-tubercolari/14390-infezione-da-mycobacterium-chimaera-nella-regione-veneto-i-pazienti-richiamati-sono-1400, accessed on 30 March 2025) and aimed to rule out an infection from MC. However, he decided to decline the invitation to participate in the screening and clinical check, citing his good clinical condition and lack of any signs or symptoms. Two years before the last admission, the patient reported fatigue, weight loss, a cough, and right-arm paresthesia coinciding with cerebral hemorrhagic lesions that were misdiagnosed as mycotic emboli and treated with corticosteroids, with the patient’s condition showing clinical improvement (Figure 1).

The combination of respiratory symptoms with hypercalcemia, elevated plasma ACE levels (123 U/L; upper limit: 64 U/L), and a high CD4/CD8 ratio raised suspicion of sarcoidosis, although a chest CT scan did not show anything remarkable or typical signs of this disease. Splenomegaly, leukopenia, and thrombocytopenia were further evaluated with peripheral lymphocyte immunophenotyping, generating suspicion of a possible hepatosplenic T lymphoma, though a bone marrow biopsy did not confirm this diagnosis.

**Figure 2:**
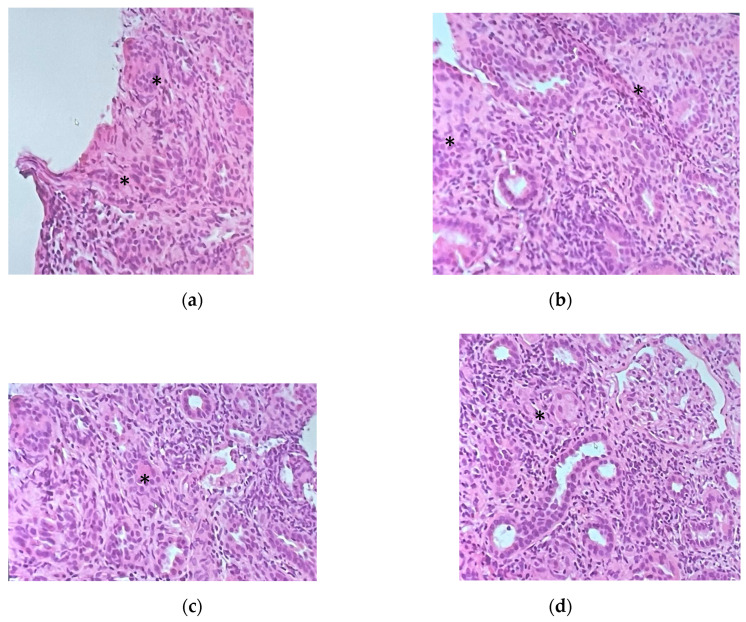
(**a**–**c**) Kidney biopsy sections showing extended lymphomonocytic cell infiltrate with rare eosinophils and granulomas with giant multinucleated cells (*). (**d**) Section showing an uninvolved glomerulus surrounded by granulomas (*) and inflammatory cell infiltrate.

Given the patient’s worsening kidney function with sub-nephrotic proteinuria and hematuria, multiple immunologic blood tests were performed, namely, ANA, ENA, ANCA, anti-GBM, and cryoglobulinemia tests, the results of which were negative in each case; the complement dosing was normal, and a monoclonal component of 1 g/L with negative Bence Jones proteinuria was detected. Since the etiology of the kidney injury remained unknown and the sCr continued to rise, a kidney biopsy was performed, revealing interstitial nephritis with eosinophils and granulomas formed by giant cells with noncaseating necrosis and a florid lymphocyte T infiltrate (CD68++, CD3+++, rare CD20 +, and CD8++, CD4+). Glomeruli (eight in total, one of which was sclerotic) appeared uninvolved (Figure 2). Immunofluorescence staining performed on four of the glomeruli revealed mild IgG (one out of eight glomeruli), C3c (two out of four), C1q (one out of four), fibrinogen (two out of four), and kappa light-chain positivity (one out of four) with a mesangial granular segmental pattern. Endothelial C3c presence was also detected. Acid-fast staining yielded a negative result. Cultures were not performed on kidney tissue. Nested-PCR genus-specific (HSP65) for Mycobacteria and PCR for Mycobacterium tuberculosis were also negative. PCR amplification of the V-J region of the gamma chain of the T-cell receptor of the lymphocytic population in the kidney biopsy showed a polyclonal result, excluding a lymphoma.

**Figure 3:**
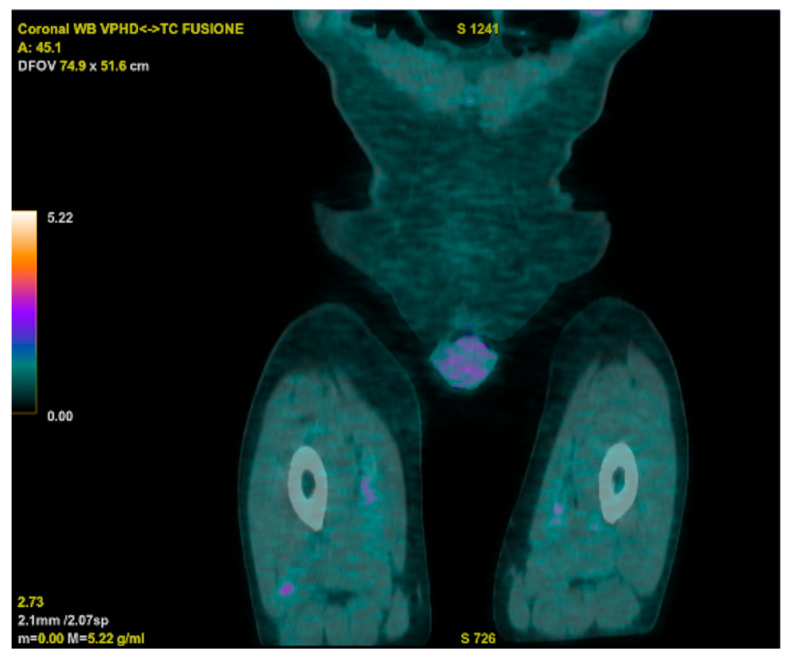
18 FDG PET-CT showing two hypercaptant lesions in the patient’s right thigh.

Meanwhile, an 18 FDG PET-CT displayed increased metabolic activity close to the prosthetic aortic valve, and a trans-esophageal echocardiogram raised suspicion of endocarditic vegetation. However, a subsequent cardiac CT ruled out vegetations but unveiled the persistence of a residual VSD. The cardiac surgeon indicated that a surgical approach was not necessary, considering the adequate functioning of the prosthetic valve. The PET-CT also revealed two hypercaptant lesions in the patient’s right thigh that were further investigated, with a biopsy showing a histiocytic inflammatory infiltrate and a specimen that was deemed suitable for microbiologic cultures (Figure 3).

Blood, urine, and broncho-aspirate cultures and extensive microbiological exams (a CMV test; an EBV test; the Interferon Gamma Release Assay (IGRA) test; Leishmania, Bartonella, Coxiella, Legionella, Mycoplasma, and Treponema pallidum tests; HCV, HIV, and HBV serologies; a serum beta D glucan test; and a galactomannan test) were all negative. At this point, blood cultures for non-tubercular Mycobacteria were still pending. The patient remained apyretic, with normal inflammation markers for the whole course of hospitalization. As these initial tests excluded infection, an empirical treatment with methylprednisolone was started, given the diagnosis of idiopathic granulomatous interstitial nephritis (GIN). We subsequently observed partial improvement of kidney function, and the patient was discharged (sent home) from the hospital with a plan for the outpatient management and follow-up.

Two weeks after the patient’s discharge, blood cultures and a culture based on a muscle biopsy came back positive for MC. The strain was shown to be sensitive to amikacin and clarithromycin, with an intermediate susceptibility to linezolid and moxifloxacin. During this time, the patient’s fever returned, and he experienced significant and worsening fatigue. Therefore, the patient was readmitted to the Infectious and Tropical Disease Division and started a combination antibiotic treatment including azithromycin (500 mg/day), moxifloxacin (400 mg/day), rifampicin (600 mg/day), and ethambutol (15 mg/kg/day), and we pursued further diagnostic exams.

A second trans-esophageal echocardiogram was performed and confirmed the presence of endocarditis vegetations. The cardiac surgery team once again excluded the need for a surgical intervention.

Meanwhile, steroid tapering was attempted, with a subsequent mild acute kidney injury relapse at first, but after two months of antimicrobial treatment, the patient’s kidney function improved to sCr 134 μmol/L.

After six weeks of treatment, a transesophageal echocardiogram disclosed a resolution of the endocarditic lesions. The patient’s blood count also improved, and a normal blood calcium level was restored. The antimicrobial regimen also led to near-complete regression of cerebral mycotic aneurysms after one month. The antimicrobial treatment was well tolerated and is still ongoing.

Together with the cardiothoracic consultants, we decided that once the blood cultures returned negative and the patient’s overall condition stabilized, a re-evaluation would take place to schedule a cardiothoracic surgery.

The detection of MC in blood and muscle biopsy cultures together with the patient’s exposure (a history of surgery requiring a cardiopulmonary bypass prior to symptoms of infection) and clinical criteria (prosthetic valve endocarditis with a bloodstream infection and a disseminated infection including embolic and immunologic manifestations) make this a confirmed case of an MC infection [7].

## 3. Discussion

GIN is a rare finding in kidney biopsies (0.5–0.9%). It is a form of interstitial nephritis, most often secondary to drug use (e.g., cotrimoxazole, methicillin, penicillin B, rifampicin, phenytoin, allopurinol, heroin, etc.), systemic immune diseases (e.g., sarcoidosis, granulomatosis with polyangiitis, etc.), and infections (especially mycobacteria and fungi) [9,10]. It is caused by a form of delayed-type hypersensitivity reaction and a cell-mediated response by type-1 helper T cells. Granulomas are aggregates of macrophages and mainly T and B cells that deposit in the interstitial space, leading to a fibrotic response that ultimately impacts organ function [5,10]. While GIN with non-necrotizing granulomas is more often drug-induced, necrotizing GIN is more common in patients with granulomatosis with polyangiitis or a fungal or tubercular infection [10]. Moreover, granulomas in sarcoidosis tend to appear more well-defined than those in drug-induced-GIN [10]. Treatment is based on the removal of the causal agent in conjunction with corticosteroid therapy [9,10].

The clinical course of our patient is similar to that presented by Trautman et al. [5]. In fact, in our case, the patient was also immunocompetent and had a history of cardiac surgery. Moreover, as in the cited case [5], there were no cultures of our patient’s kidney tissue; only acid-fast staining was ordered, the results of which came back negative. However, in our case, mycobacterium genus-specific PCR (HSP-65) was performed on the kidney tissue, as recommended by international guidelines that suggest that *Mycobacterium* genus-specific PCR should be considered if histopathology shows non-caseating granulomas and foamy swollen macrophages with or without acid-fast bacilli (Class IIa, Level C”) [7]. A PCR targeting HSP65 generally shows higher sensitivity compared to other PCRs conducted on paraffin-embedded specimens [7], but (to the best of our knowledge) kidney tissue has never been evaluated before [11]. We therefore speculate that the granulomatous interstitial nephritis investigated herein, which yielded negative results regarding acid-fast staining and mycobacteria PCR investigations, suggests that, when considering the glomerular immunofluorescence pattern, as in Trautman et al.’s case, the kidney involvement observed was arguably more likely an immunogenic response to the infection rather than a direct renal MC infection [5]. The same authors indicated that the presence of AFB within the granulomas of interstitial nephritis should be expected more in immunocompromised patients than in immunocompetent ones [5,6]. In our case, blood cultures reported the growth of MC only two weeks after the negative results of the mycobacteria PCR testing of the kidney biopsy, contributing to a misleading diagnosis and thus corticosteroid prescription, delaying the initiation of an appropriate antimicrobial therapy.

A peculiar aspect of our case is the presentation of hypercalcemia and suppressed PTH, which also contributed to the initial suspicion of sarcoidosis. In non-tubercular-mycobacteria infections, hypercalcemia may be due to continuous hydroxylation of vitamin D by the alpha-hydroxylase independently produced by granulomas’ macrophages [12]. To the best of our knowledge, hypercalcemia has never been reported in patients with an active MC infection, but it has been described as clinical sign of a rare Immune Reconstitution Inflammatory Syndrome (IRIS) only after the treatment of MC-induced mediastinitis [13]. Instead, our patient presented with hypercalcemia that improved after antimicrobial therapy initiation.

As in previously described cases [5,6], the misdiagnosis of the granulomatous interstitial nephritis led to corticosteroid treatment that, despite improving kidney function, worsened the infection. Therefore, we agree with Trautman et al.’s suggestion that for patients with granulomatous interstitial nephritis with no observable AFB in a kidney biopsy and a history of cardiac surgery, it is advisable to forego corticosteroid treatment, as mycobacteria cultures may turn positive later, and steroids could worsen the patients’ outcomes [5].

In cases of a suspected MC infection, echocardiography is crucial in the diagnostic work-up [7]. Trans-esophageal echocardiography offers higher sensitivity compared to transthoracic echocardiography and is considered the gold standard for the detection of cardiac vegetations and aortic root collections and the assessment of valvular function (Class I, Level C) [7]. In regard to our patient, during his first hospitalization in the Nephrology Unit, a PET-CT scan showed mildly increased metabolic activity close to the prosthetic aortic valve, and the patient underwent trans-esophageal echocardiography that revealed the presence of a moving mass adherent to the aortic prosthesis that was judged to be either a thrombotic lesion or an endocarditis vegetation. A cardiac CT was then ordered by the cardiologist to resolve the uncertainty, ultimately excluding vegetations adherent to the prosthesis. This discrepancy between different imaging techniques contributed to the delay in the diagnosis of endocarditis. Only subsequent transthoracic echocardiography, performed when the patient showed septic signs, removed doubts about the diagnosis of endocarditis. Additionally, our patient had already been diagnosed with mycotic aneurysms two years prior to his first hospitalization: had this finding been further investigated with echocardiography at that time, the diagnosis of endocarditis could have been made two years earlier. Multidisciplinary management was crucial in this case.

Finally, the myositis manifestation is rare in disseminated MC infections [2]. To the best of our knowledge, our patient is only the second reported case of muscle abscess with a positive culture for MC [14].

In conclusion, our case report underlines the importance of suspecting MC infection in patients with granulomatous interstitial nephritis who have undergone cardiac surgery, even when mycobacteria are not isolated from a kidney biopsy, in order to prevent delayed diagnosis and treatment initiation.

## Data Availability

The data presented in this study are available on request from the corresponding author due to privacy and ethical restrictions.
